# First records of the genus *Pelionella* Kaydan, 2015 in East Asia, with description of a new species (Hemiptera, Coccomorpha, Pseudococcidae)

**DOI:** 10.3897/zookeys.738.13277

**Published:** 2018-02-19

**Authors:** Hirotaka Tanaka

**Affiliations:** 1 Faculty of Agriculture, Ehime University, Tarumi 3-5-7, Matsuyama, Ehime 790-8566, Japan

**Keywords:** *Artemisia indica*, Japanese mugwort, new distribution record, taxonomy

## Abstract

Two mealybug species (Hemiptera: Coccomorpha: Pseudococcidae), *Pelionella osakaensis* **sp. n.** and *P. manifecta* (Borchsenius, 1949), are described and illustrated based on adult female specimens collected in Japan, on the Japanese mugwort Artemisia indica Willd. var. maximowiczii (Nakai) H. Hara (Asteraceae). These are the first records of the occurrence of *Pelionella* species in East Asia. The new species is similar to *P. grassiana* (Goux, 1989) and *P. proeminens* (Goux, 1990), but differs in lacking multilocular pores with double loculi rings on the venter and in possessing dorsal cerarii and a circulus. The Japanese population of *P. manifecta* is morphologically slightly different from the Azerbaijani and French populations in lacking large-type oral-collar tubular ducts associated with clusters formed by multilocular pores and oral-collar ducts on ventral abdominal segments III and IV. A modified key to species of the genus *Pelionella* Kaydan, 2015, is provided.

## Introduction

A genus of Pseudococcidae (Hemiptera: Coccomorpha), *Pelionella* Kaydan, 2015, was erected by Kaydan (2015) for a genus related to *Peliococcus* Borchsenius, 1948, based on molecular and morphological analyses. Kaydan (2015) defined the genus *Pelionella* based mainly on three diagnostic characters: (i) the presence of a special type of multilocular pore, consisting of double rings of eleven loculi, mainly located within pore clusters on the dorsum; (ii) the absence of a type of dorsal seta similar to cerarian setae that lacks a trilocular pore near the basal socket and is not located on an elevated area; and (iii) the presence of one or two sizes of dorsal oral-collar tubular ducts, and smaller ducts present in the center of clusters of multilocular pores with double rings on the dorsum. To date, eleven species of the genus have been recorded from the Western Palearctic and Eastern Nearctic (García Morales et al. 2016; Kaydan 2015) but none from East Asia, including Japan, although some species of the genus *Peliococcus* have been recorded (Kwon et al. 2003; Tanaka 2017; Tang 1992). *Peliococcus* and *Pelionella* can be clearly recognized by some morphological differences, such as presence or absence of a special type of multilocular pore consisting of double loculi rings, and clusters formed by the pores and several types of oral-collar ducts mainly on the dorsal surface.

Recently, the author examined specimens of two species of *Pelionella* collected from Japan, and recognized among the samples the type species of the genus, *Pelionella manifecta* (Borchsenius, 1949), and a single specimen of an undescribed species. The former showed slight differences from western populations in some morphological character states. This paper describes or diagnoses and illustrates both species collected from Japan based on adult female morphology, and constitutes the first record of the occurrence of *Pelionella* species in East Asia. A modified key to species of the genus *Pelionella* is also given.

This new distribution record for *P. manifecta*, and the description of a new species of *Pelionella* with unique morphological features (i.e., presence of several dorsal cerarii distinctly elevated from dorsal surface) may be useful for understanding and furthering studies on the diversity, morphology, and biogeography of this genus and other related mealybug species.

## Materials and methods

Examined materials were collected by I. Takahashi, J. Imai, or K. Fujimoto in the fall (from October to November) of 2014. The adopted slide-mounting method is a slight modification of Kawai (1980) which uses lemosol (95% limonen) as a substitute for xylene. Slide-mounted specimens were examined under a phase-contrast compound microscope (Olympus BH2-PH, Tokyo, Japan). The description format and morphological terminology mostly follow Kaydan (2015). The holotype material of the new species and the voucher specimens of *P. manifecta* examined in this study are deposited in the National Museum of Nature and Science, Tsukuba, Japan (**NSMT**). The remaining materials of *P. manifecta* examined and used for description in this study are deposited in the Dr. Kawai scale insect collection in Tokyo University of Agriculture.

## Taxonomy

### Genus Pelionella

Taxon classificationAnimaliaHemipteraPseudococcidae

Kaydan, 2015

Pelionella Kaydan, 2015: 226.
Pelionella ; Danzig & Gavrilov-Zimin, 2014: 449 (Unavailable name).

#### Type species.

*Peliococcus manifectus* Borchsenius, 1949: 245.

### Pelionella manifecta

Taxon classificationAnimaliaHemipteraPseudococcidae

(Borchsenius, 1949)

Figure 1

Peliococcus manifectus Borchsenius, 1949: 245: Danzig, 2001: 125.
Peliococcus albertaccius Goux, 1990: 83.
Pelionella manifecta (Borchsenius, 1949); Danzig and Gavrilov-Zimin 2014: 457 (as an unavailable name); Kaydan 2015: 234.

#### Material studied.

All three adult females from Japan collected on Artemisia indica Willd. var. maximowiczii (Nakai) H. Hara (Asteraceae). Osaka-pref., Sennan City, Kansai International Airport, on: 1 adult female, 7.X.2014, coll. I. Takahashi; 1 adult female, 12.X.2014, coll. K. Fujimoto. Hyogo-pref., Kobe City, Chuo-ku, Minato-jima, Naka-machi: 1 adult female, 8.XI.2014, coll. J. Imai (NSMT-I-Ho 00082).

#### Description.

**Slide-mounted specimens of Japanese populations, n = 3.** *Adult female.* Body elongate oval, 1.7–3.1 mm long, 0.9–1.8 mm wide. Eyes on margins, each 31–44 μm in diameter. Antenna 9-segmented, 393–444 μm long; apical segment 58–60 μm long, 22–25 μm wide; with two apical setae each 36–45 μm long, and three fleshy setae 18–30 μm long. Labium 95–110 μm long, 80–88 μm wide. Circulus oval, 95–100 μm wide, situated just anterior to fold between abdominal segments III and IV. Legs well developed; hind legs: coxa 120–140 μm long; trochanter + femur 248–282 μm long; tibia + tarsus 285–306 μm long; claw 30–32 μm long; translucent pores absent. Ratio of lengths of tibia + tarsus to trochanter + femur 1.05–1.18:1; ratio of lengths of tibia to tarsus 2.0–2.41:1; ratio of length of trochanter + femur to greatest width of femur 3.22–4.10:1. Tarsal digitules hair-like, each 23–30 μm long. Claw digitules knobbed, each 25–29 μm long. Claw with well-developed denticle on plantar surface. Anterior ostioles each with a total for both lips of 15–25 trilocular pores and 2–5 setae; posterior ostioles each with a total for both lips of 24–37 trilocular pores and 4–7 setae. Anal ring 73–108 μm wide, bearing 6 setae, each seta 127–190 μm long.

*Dorsum.* Setae spine-like, each 5–15 μm long. Cerarii on margin somewhat prominent, slightly sclerotized, numbering 18 pairs; anal lobe cerarii each with 1–2 slender enlarged setae, each 10–22 μm long, and one or two spine-like auxiliary setae; other cerarii mostly each with two enlarged setae and several trilocular pores. Clusters of multilocular pores with double rings present on head and thorax and on abdominal segments as follows: I 9–12, II 12, III 14–15, IV 16–19, V 18–22, VI 10–15, VII 9–11, VIII 0; each cluster containing 1–7 (usually 2-3) multilocular pores with double rings, each pore 6.0–7.1 μm in diameter; a small oral-collar tubular duct, 0.5–1.8 μm wide; 1–5 large oral-collar tubular ducts, each 2.4–3.4 μm wide; and 1–3 minute discoidal pores, each 1.1–1.3 μm in diameter. Trilocular pores, each 3.2–3.9 μm in diameter, scattered throughout. Minute discoidal pores mainly restricted to within clusters.

*Venter.* Setae of two types: (i) slender hair-like setae, each 10–142 μm long, longest setae situated medially on head; and (ii) spine-like setae in submarginal areas, each 4–12 μm long. Apical setae of anal lobes 198–228 μm long. Multilocular disc pores with single ring, each 5.0–6.8 μm in diameter, present in 15–25 clusters on medial areas of abdominal segments III and IV; each cluster containing 1–5 (usually 2–3) multilocular disc pores surrounding a small oral-collar tubular duct; similar multilocular disc pores present also in single rows on other abdominal segments, as follows: V 7–8, VI 43–47, VII 62–69, VIII + IX 38–46. Multilocular pores with double rings, each 6.6–7.9 μm in diameter, restricted to submarginal areas of head, thorax, and abdomen, usually not arranged in clusters. Quinquelocular pores, each 3.2–5.6 μm in diameter, scattered medially on head, thorax, and medial area of abdominal segments. Trilocular pores, each 2.6–3.2 μm in diameter, scattered throughout. Minute discoidal pores, each 0.8–1.3 μm in diameter, few. Oral-collar tubular ducts of two sizes: small ducts restricted to within clusters; and large-sized ducts, each 2.1–2.9 μm wide, present on body margin and in single rows across posterior abdominal segments; also a few on head, thorax and abdominal segments II and III.

**Figure 1.**
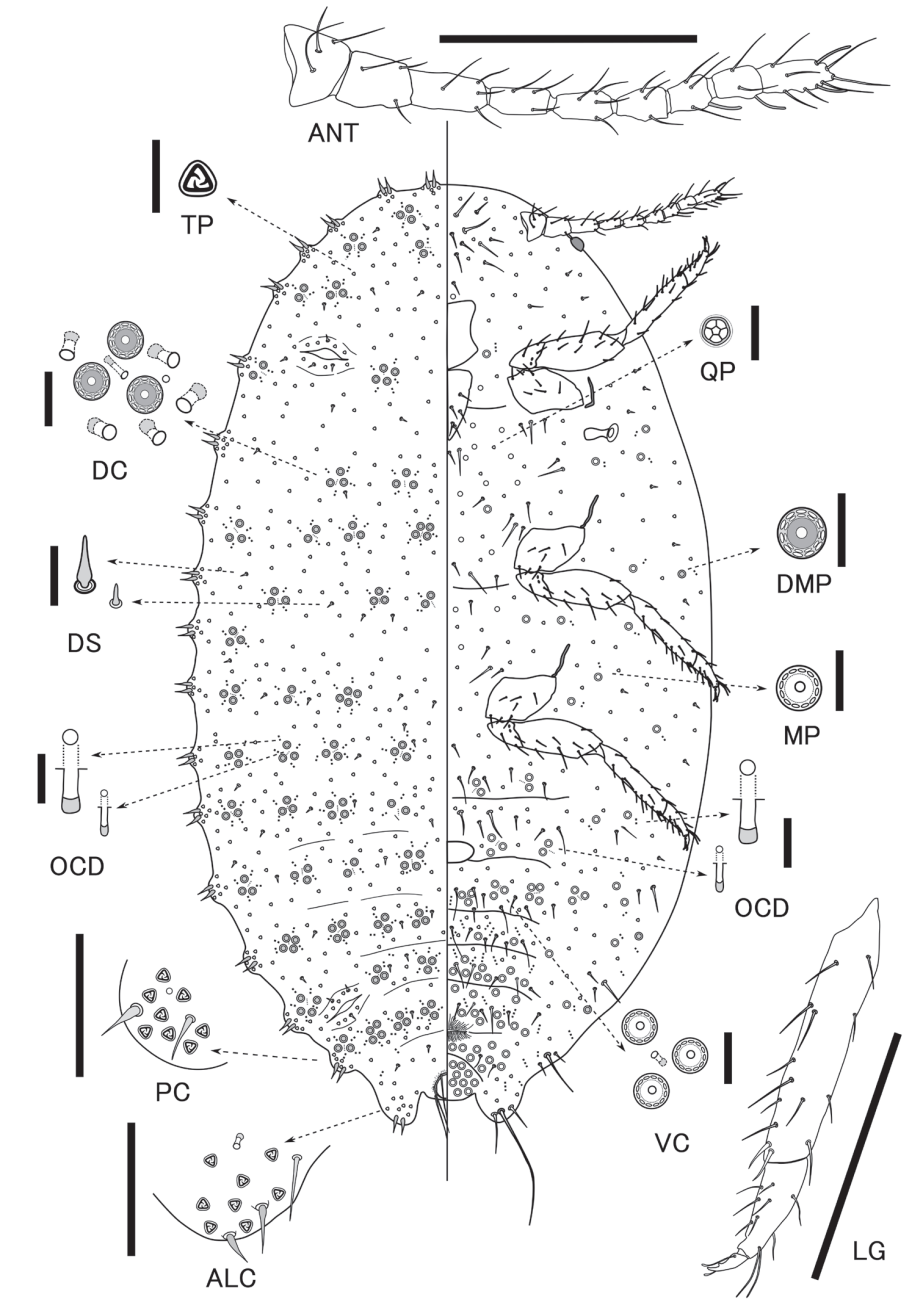
*Pelionella manifecta* (Borchsenius, 1949) collected in Japan. Adult female. Abbreviations: **ALC**, anal lobe cerarius **ANT** antenna **DC** dorsal cluster **DS** dorsal setae **DMP** Multilocular pore with double rings **LG** leg **MP** multilocular pore **OCD** oral-collar tubular ducts **PC** Penultimate cerarius **QP** quinquelocular pore **TP** trilocular pore **VC** ventral cluster. Scale bars: 200 µm (**ANT, LG**); 50 µm (**ALC, PC**); 10 µm for the others.

#### Distribution.

Armenia, Azerbaijan, Corsica, France, Italy, Kazakhstan, Russia (Krasnodar Territory), Sardinia, Sweden, Turkey (Kaydan 2015), and Japan.

#### Discussion.

The Japanese specimens of *Pelionella manifecta* described here differ slightly from the Azerbaijani and French material described by Kaydan (2015) in having many more multilocular disc pores on the venter of the abdominal segments, much smaller tubular ducts and pores (Table 1) and in lacking the large oral-collar tubular ducts associated with multilocular pore clusters on venter of abdominal segments III and IV. However, these morphological differences are herein tentatively regarded as intraspecific variation, because the number of tubular ducts and multilocular pores is known to vary greatly in some mealybug species (Cox 1983; Charles et al. 2000; Chatzidimitriou et al. 2016), considerable geographical morphological variation within *P. manifecta* has been also recorded (Kaydan 2015), and hitherto, the morphological variation of *P. manifecta* and taxonomic significances of the ducts’ and pores’ sizes have not been sufficiently studied. This description of the Japanese population may be useful for understanding phenotypic variation in the species. Future molecular studies may help elucidate the extent of variation in *P. manifecta*.

In Japan, this species was collected from Kansai International Airport, one of the largest airports in the country, and from the large sea-port island of Kobe City (Minato-jima), both of which are centres of international trade. Furthermore, the species has not hitherto been recorded further east than Kazakhstan. This suggests that the species might not be truly endemic to Japan, but be a recent introduction. Studies of the detailed distribution of the species in Japan, and the current condition of the species at the sites where it was collected originally, may be important from both biological and plant-quarantine perspectives.

**Table 1.** Comparisons of morphometric data between adult females of Japanese and Western Eurasian populations of *Pelionella manifecta*.

**Morphological features**	**Measurements of Japanese specimens (n = 3)**	**Measurements of Azerbaijani and French specimens (from Kaydan, 2015) (n = 5)**
**General morphological features**		
Length of body	1.7–3.1 mm	1.36–1.88 mm
Width of body	0.9–1.8 mm	0.86–1.10 mm
Width of eyes	31–44 µm	47.5–60.0 µm
Lendth of antenna (total)	393–444 µm	410–425 µm
Length of antenna’s apical segment	58–60 µm	60 µm
Width of antenna’s apical segment	22–25 µm	20–28 µm
Length of antenna’s apical setae	36–45 µm	27–45 µm
Length of fleshly setae on antenna’s apical segment	18–30 µm	25–33 µm
Length of labium	95–110 µm	135–140 µm
Width of labium	80–88 µm	95 µm
Width of circulus	95–100 µm	65–85 µm
Length of hind coxa	120–140 µm	155–175 µm
Length of hind trochanter and femur	248–282 µm	240–260 µm
Length of hing tibia and tarsus	285–306 µm	260–280 µm
Length of hind claw	30–32 µm	25–30 µm
Ratio of lengths of hind tibia + tarsus to hind trochanter + femur	1.05–1.18:1	1.07–1.23:1
Ratio of lengths of hind tibia to hind tarsus	2.0–2.41:1	2.16–2.41:1
Ratio of length of hind trochanter + femur to greatest width of hind femur	3.22–4.10:1	3.42–4.0:1
Length of hind tarsal digitules	23–30 µm	20–23 µm
Length of hind claw digitules	25–29 µm	20–25 µm
**Morphological features on Dorsum**		
The number of triolocular pores on anterior ostiole	15–25	21–30
The number of setae on anterior ostiole	2–5	2–4
The number of triolocular pores on posterior ostiole	24–37	32–40
The number of setae on posterior ostiole	4–7	2–4
Width of anal ring	73–108 µm	85–110 µm
Lenth of anal ring setae	127–190 µm	115–145 µm
Length of anal lobe cerarian setae	10–22 µm	17–23 µm
The number of auxiliary setae on anal lobes	1–2	3–4
Length of dorsal setae	5–15 µm	7.5–15 µm
The number of multilocular pore with double rings in clusters	1–7	2–6
Width of multilocular pores with double rings in clusters	6.0–7.1 µm	7.5–10.0 µm
Width of small oral collar tubular ducts in clusters	0.5–1.8 µm	3.0–4.0 µm
Width of large oral collar tubular ducts in clusters	2.4–3.4 µm	4.0–5.0 µm
The number of minute discoidal pores in clusters	1–3	1–4
Width of minute discoidal pores in clusters	1.1–1.3 µm	2 µm
The number of dorsal clusters in abdominal segment I.	9–12	9–11
The number of dorsal clusters in abdominal segment II.	12	10
The number of dorsal clusters in abdominal segment III.	14–15	11–13
The number of dorsal clusters in abdominal segment IV.	16–19	11–15
The number of dorsal clusters in abdominal segment V.	18–22	12
The number of dorsal clusters in abdominal segment VI.	10–15	8–11
The number of dorsal clusters in abdominal segment VII.	9–11	10–14
Width of triolocular pores	3.2–3.9 µm	3–5 µm
**Morphological features on Venter**		
Length of slender hair-like setae	10–142 µm	15–88 µm
Length of spine-like setae	4–12 µm	10.0–12.5 µm
Length of apical setae on anal lobes	198–228 µm	145–185 µm
Width of multilocular pores with double rings	6.6–7.9 µm	7.5–10.0 µm
Witdth of multilociular pores with single ring	5.0–6.8 µm	7.5–10.0 µm
The number of clusters on abdominal segments III-IV	15–25	10–14
The number of multilocular pores on abdominal segment V.	7–8	2–3
The number of multilocular pores on abdominal segment VI.	43–47	14–18
The number of multilocular pores on abdominal segment VII.	62–69	34–40
The number of multilocular pores on abdominal segments VIII+IX.	38–46	20–23
Width of quinquelocular pores	3.2–5.6 µm	5.0–7.5 µm
Width of triolocular pores	2.6–3.2 µm	2–3 µm
Width of minute discoidal pores	0.8–1.3 µm	2 µm

### Pelionella osakaensis sp. n.

Taxon classificationAnimaliaHemipteraPseudococcidae

http://zoobank.org/F2AB23EA-CD3B-4CB1-938A-DED7C5F714FE

Figure 2

#### Holotype.

Adult ♀. Japan, Osaka-pref., Sennan City, Kansai International Airport, 12.X.2014, host plant: Artemisia indica var. maximowiczii, coll. K. Fujimoto. (NSMT-I-Ho 00081).

#### Diagnosis.

Eighteen pairs of cerarii present on body margin. Several slightly elevated dorsal cerarii also present on dorsal surface. Clusters of multilocular pores with double rings present on dorsum; each cluster contains 1–2 multilocular pores with double rings, 0–1 small oral-collar tubular ducts, 0–2 large oral-collar tubular ducts, and 0–3 minute discoidal pores. Multilocular pores with double rings and clusters of multilocular disc pores with single ring and oral-collar tubular ducts absent on venter. Circulus oval, present on posterior part of third abdominal segment of venter. Translucent pores absent on hind legs.

#### Description.

**Slide-mounted specimen.** *Adult female.* Body elongate oval, 1.7 mm long, 0.9 mm wide. Eyes submarginal, each 30–32 μm in diameter. Antenna 9-segmented, 363–387 μm long; apical segment 53–57 μm long, 20–25 μm wide; with two apical setae each 30–38 μm long, and three fleshy setae each 20–30 μm long. Labium 103 μm long, 68 μm wide. Circulus oval, approx. 74 μm wide, situated on posterior part of third abdominal segment. Legs well developed; posterior legs: coxa 82–85 μm long; trochanter + femur 248–250 μm long; tibia + tarsus 275–278 μm long; claw 30–34 μm long. Translucent pores absent. Ratio of lengths of hind tibia + tarsus to trochanter + femur 1.1:1; ratio of lengths of tibia to tarsus 1.4–1.5:1; ratio of length of trochanter + femur to greatest width of femur 3.0:1. Tarsal digitules hair-like, each 20–31μm long. Claw digitules knobbed, each 28 μm long. Claw with well-developed denticle on plantar surface. Anterior ostioles with a total for both lips of 26 to 30 trilocular pores and 2–3 setae; posterior ostioles with a total for both lips of 33–36 trilocular pores and 5–6 setae. Anal ring 85 μm wide, bearing 6 setae, each seta 103–125 μm long.

**Figure 2.**
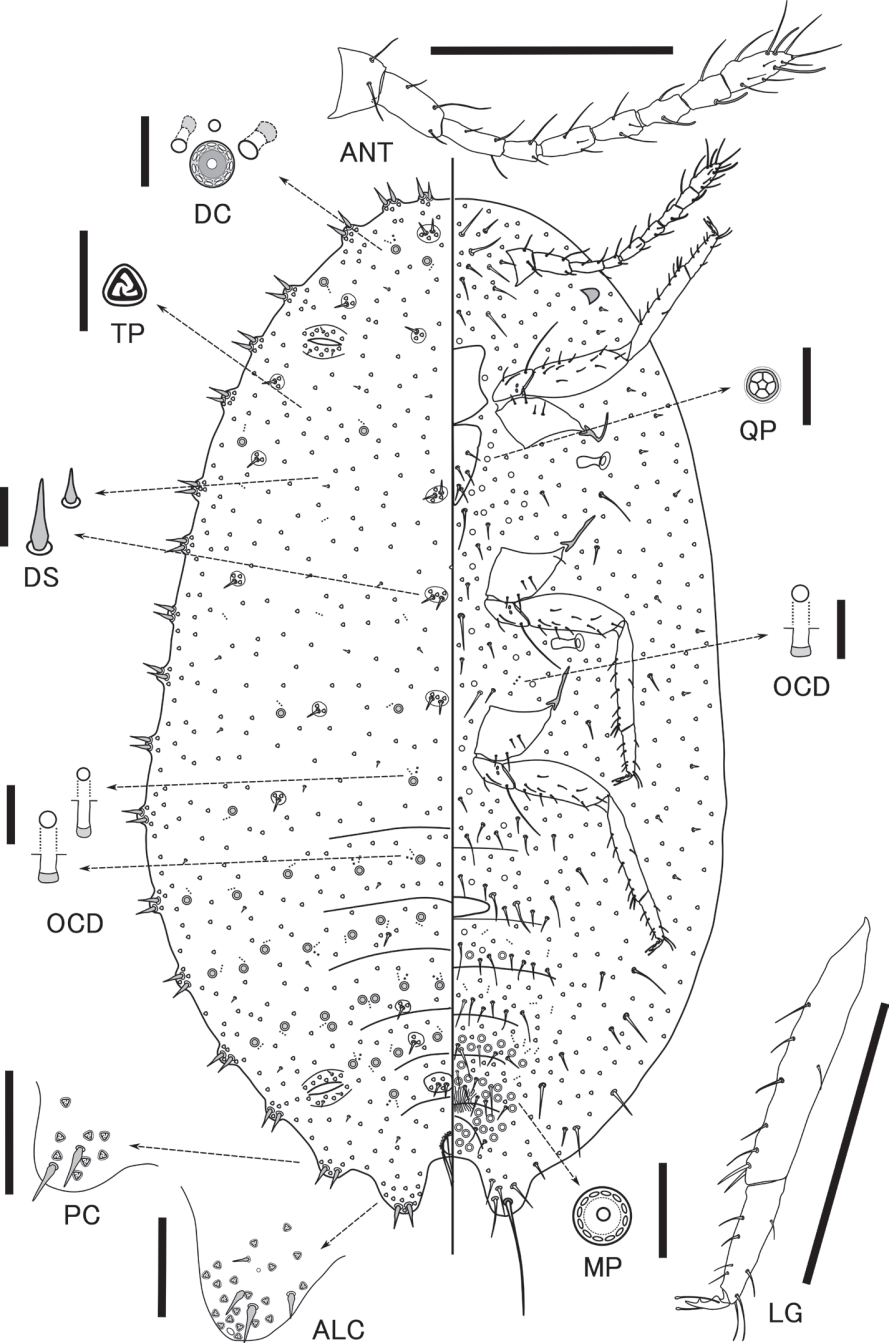
*Pelionella osakaensis* sp. n. adult female (holotype). **ALC**, anal lobe cerarius **ANT** antenna **DC** dorsal cluster **DS** dorsal setae **LG** leg **MP** multilocular pore **OCD** oral-collar tubular ducts **QP** quinquelocular pore **PC** Penultimate cerarius **TP** trilocular pore. Scale bars: 200 µm (**ANT, LG**); 50 µm (**ALC, PC**); 10 µm for the others.

*Dorsum.* Cerarii on margins slightly prominent but with no sclerotization, numbering 18 pairs; anal lobe cerarii each with 2–3 slender enlarged setae, each 11–21 μm long, and 2–3 spine-like auxiliary setae; other cerarii on margins each with 2–3 slender enlarged setae and several trilocular pores. Several dorsal cerarii present on dorsal surfaces as shown in Figure 2, each cerarii with 1–2 relatively small enlarged setae, no auxiliary setae and several triolocular pores; each slightly prominent but with no sclerotization. Setae spine-like, each 5–15 μm long; larger setae each with 1–3 trilocular pores near base, sometimes forming dorsal cerarii. Multilocular pores with double rings, each 5.5–6.2 μm in diameter, in each cluster present singly or in pairs together with 0–1 small oral-collar tubular ducts, 1.8–2.0 μm wide, plus 0–2 large oral-collar tubular ducts, each 2.5–2.8 μm wide, and 0–3 minute discoidal pores, each 1.2–1.8 μm in diameter. Clusters few on head and thorax, and present on abdominal segments as follows: I 4, II 4, III 11, IV 11, V 13, VI 2, VII 3, VIII+IX 0. Trilocular pores, each 3.2–3.8 μm in diameter, scattered throughout. Several small oral-collar ducts, each 1.8–2.0 μm wide, and minute discoidal pores, each 1.2–1.8 μm in diameter, sometimes present outside the clusters.

*Venter.* Setae of two types: (i) hair-like setae, each 14–81 μm long, longest present on medial area of posterior abdominal segments; and (ii) spine-like setae, each 5–10 μm long, present in submarginal areas. Apical setae of anal lobes each 162–169 μm long. Multilocular disc pores, each 5.5–6.5 μm in diameter, present in bands on abdominal segments as follows: IV 4, V 0, VI 28, VII 34, VIII + IX 29. Quinquelocular pores, each 3.5–4.5 μm in diameter, scattered medially on head, thorax, and first four abdominal segments. Trilocular pores, each 2.8–3.2 μm in diameter, scattered throughout. Minute discoidal pores, each 1.0–1.2 μm in diameter, few in number. Oral-collar tubular ducts of 1size, each 1.9–2.1 μm wide, mostly present in bands across posterior abdominal segments and on medial areas of thoracic segments; a few ducts present in submarginal areas.

#### Etymology.

The species is named after the prefecture in Japan where it was collected.

#### Discussion.

*Pelionella osakaensis* sp. n. is quite similar to *P. grassiana* (Goux, 1989) and *P. proeminens* (Goux, 1990) in having clusters containing one or two multilocular pores with double rings on dorsum and more than 16 pairs of cerarii. However, *P. osakaensis* differs from the latter species in having a circulus on the posterior part of the third abdominal segment, several slight elevated dorsal cerarii, and in lacking translucent pores on hind legs. Although the presence or absence of a circulus can be variable within a mealybug species, it may be a useful, readily observable diagnostic character for *P. osakaensis* given the current status of classification of *Pelionella* species. *Pelionella osakaensis* is also similar to *P. stellarocheae* (Goux, 1990) in lacking translucent pores on hind legs and in having smaller number of multilocular pores with double rings in each cluster on dorsum; however, it clearly differs from *P. stellarocheae* in having 18 pairs of cerarii plus dorsal cerarii. The presence of dorsal cerarii is one of the important features of *P. osakaensis*, although it may appear to conflict with the generic definition of *Pelionella* proposed by Kaydan (2015). Here the importance of the presence of multilocular pores with double rings in the clusters is emphasized, so the new species is considered to belong to the genus *Pelionella*. Further research into the generic definition of the genus *Pelionella* is still much needed.

*Pelionella osakaensis* has only been collected from the site of Kansai International Airport, one of the largest airports in Japan, so it is possible that it is not endemic. A more detailed distributional study of the species and the current population level and distribution of the species at the airport may be important in relation to plant-quarantine measures.

##### Key to species of the genus *Pelionella* based on adult female morphology (adopted and partially modified from Kaydan 2015)

**Table:** 

1	Clusters on dorsum each with only 1 size of oral-collar tubular duct; multilocular pores with double rings around cluster without larger oral-collar tubular ducts	**2**
–	Clusters on dorsum each with 2 sizes of oral-collar tubular ducts, with smaller ducts in center of each cluster and larger ducts and multilocular pores with double rings around cluster (very rarely multilocular pores with double rings few on dorsum but ducts still in clusters, i.e., *P. glandulifer* (Borchsenius) and *P. tritubulata* (Kiritchenko)	**3**
2	With 3–6 (generally fewer than 5) multilocular pores with double rings in each cluster on thorax and head; each anal lobe cerarius with 4 enlarged cerarian setae	***P. balteata* (Green, 1928)**
–	With 4–8 (generally more than 5) multilocular pores with double rings in each cluster on thorax and head; each anal lobe cerarius with only 2 enlarged setae and 2 smaller auxiliary setae	***P. cycliger* (Leonardi, 1908)**
3	Circulus present	**4**
–	Circulus absent	**10**
4	Quinquelocular pores numerous on venter; marginal cerarii numbering 13–18 pairs	**5**
–	Quinquelocular pores absent or, if a few present, mainly around mouthparts; fewer than 8 pairs marginal cerarii	**9**
5	Each dorsal cluster with 0–4 multilocular pores with double rings and 0–3 large oral-collar tubular ducts	**6**
–	Each dorsal cluster with 2–16 multilocular pores with double rings, and 2–13 large oral-collar tubular ducts	**7**
6	Clusters on dorsum few (3–5 in total); each cluster normally without multilocular pores with double rings but with 1–3 large oral-collar tubular ducts around a central smaller duct	***P. tritubulata* (Kiritchenko, 1940)**
–	Clusters abundant throughout dorsum, each cluster with 1–3 multilocular pores with double ring, 0–3 large oral-collar tubular ducts and 0–1 small oral-collar tubular duct	**8**
7	Each cluster with 5–16 (usually 8–10) multilocular pores with double rings, 5–13 large oral-collar tubular ducts, and 7–9 minute discoidal pores; quinquelocular pores extremely sparse on venter	***P. multipora* Kaydan, 2015**
–	Each cluster with 1–7 (usually 2–4) multilocular pores with double rings, 1–5 large oral-collar tubular ducts, and 1–4 minute discoidal pores; quinquelocular pores common on venter	***P. manifecta* (Borchsenius, 1949)**
8	Dorsal cerarii absent. Cerarii numbering 13 pairs	***P. stellarocheae* (Goux, 1990)**
–	Dorsal cerarii present. Cerarii numbering 18 pairs	***P. osakaensis* sp. n.**
9	Multilocular pores with double rings on dorsum generally absent; if present, very few, restricted to posterior abdominal segments; each cluster with 0 or 2 (usually 0) multilocular pores with double rings, 1–4 large oral-collar tubular ducts, and 2–4 minute discoidal pores	***P. glandulifer* (Borchsenius, 1949)**
–	Multilocular pores with double rings present in clusters on dorsum, each cluster with 2–5 (usually 3) multilocular disc pores, 2–5 large oral-collar tubular ducts, and 2–4 minute discoidal pores	***P. kansui* Kaydan, 2015**
10	Marginal cerarii numbering 14–18 pairs; multilocular disc pores restricted to abdominal segments VI–VIII	**11**
–	Marginal cerarii numbering fewer than 4 pairs; multilocular disc pores present on abdominal segments IV–VIII	***P. sablia* (Goux, 1989)**
11	Clusters on dorsum common and in distinct rows on each segment; femur without translucent pores	***P. grassiana* (Goux, 1989)**
–	Clusters on dorsum sparsely distributed on each segment, not forming distinct rows; femur with translucent pores	***P. proeminens* (Goux, 1989)**

## Supplementary Material

XML Treatment for Pelionella

XML Treatment for Pelionella manifecta

XML Treatment for Pelionella osakaensis
